# Water-saving techniques: physiological responses and regulatory mechanisms of crops

**DOI:** 10.1007/s44307-023-00003-7

**Published:** 2023-10-26

**Authors:** Yu Chen, Ya-Nan Leng, Fu-Yuan Zhu, Si-En Li, Tao Song, Jianhua Zhang

**Affiliations:** 1 State Key Laboratory of Tree Genetics and Breeding, Co-Innovation Center for Sustainable Forestry in Southern China, Key Laboratory of State Forestry and Grassland Administration on Subtropical Forest Biodiversity Conservation, College of Life Sciences, Nanjing Forestry University, Nanjing, 210037 China; 2 Center for Agricultural Water Research in China, China Agricultural University, Beijing, China; 3 Department of Biology, Hong Kong Baptist University, and State Key Laboratory of Agro-Biotechnology, Chinese University of Hong Kong, Hong Kong, 999077 China

**Keywords:** Water-saving irrigation technology, Water use efficiency, Crops, Molecular regulatory, Adaptive growth, Environment

## Abstract

Water-saving irrigation techniques play a crucial role in addressing water scarcity challenges and promoting sustainable agriculture. However, the selection of appropriate water-saving irrigation methods remains a challenge in agricultural production. Additionally, the molecular regulatory mechanisms of crops under water-saving irrigation are not yet clear. This review summarizes the latest research developments in the application of different water-saving irrigation technologies to five important crops (rice, wheat, soybeans, maize, and cotton). It provides an overview of the impact of different irrigation techniques on crop yield, water use efficiency (WUE), physiology, growth, and environmental effects. Additionally, the review compares and contrasts the molecular regulatory mechanisms of crops under water-saving irrigation techniques with those under traditional drought stress, emphasizing the significance of combining irrigation technologies with genetic engineering for developing drought-resistant varieties and improving WUE. Furthermore, the integration of various technologies can stimulate new management strategies, optimize water resource utilization, and enhance sustainability, representing a major focus for future research. In conclusion, this review underscores the importance of water-saving irrigation technologies, especially when combined with genetic engineering, in addressing water resource scarcity, increasing crop yields, and promoting sustainable agriculture.

**Supplementary Information:**

The online version contains supplementary material available at 10.1007/s44307-023-00003-7.

## Introduction

Irrigation is the largest consumer of water in human society and an important means of increasing crop yields and mitigating the impact of drought (Wang et al. 2021a). However, irrigation water is a scarce and expensive resource, and the scarcity of water resources poses a serious threat to sustainable agriculture. This has led to an increased demand for water-saving technologies (Rao et al. 2016; Cheng et al. 2021a). The level of irrigation is closely related to crop yield and water use efficiency (WUE), which is also known as water productivity (WP) when used for yield (Tong et al. 2022; García-Tejera et al. 2018; Bozkurt Çolak 2021). Currently, drought and water stress are the main causes leading to a decrease in crop yield and quality (Tong et al. 2022; García-Tejera et al. 2018; Bozkurt Çolak 2021; Chen et al. 2021). The challenge lies in striking a delicate balance between meeting the water needs of crops and conserving this precious resource for sustainable use. In recent years, water-saving irrigation technologies have gradually become a crucial solution to address the conflict between agricultural production and water usage (Chen et al. 2023). Today, full irrigation (FI) and deficit irrigation (DI) are the two main irrigation strategies, with the former requiring sufficient water to irrigate plants and the latter allowing for a certain amount of water deficit (Peake et al. 2016; Wen et al. 2018). To meet diverse water-saving requirements and cater to different crops, these two distinct irrigation strategies (FI and DI) have given rise to various specific water-saving irrigation techniques. Simultaneously, after conducting a keyword co-occurrence network analysis and burst word detection analysis in the Web of Science Core Collection database, it was observed that water-saving irrigation techniques in crops are closely associated with WUE, grain yield, treatment, and farmers, and their importance is steadily increasing (Figure S1). Moreover, the data show that among these key crops, rice, wheat, soybean, maize, and cotton are at the forefront. These five crops are also the most widely grown globally, providing sustenance for the majority of the world's population (Hergert et al. 2016). Therefore, investigating the impact of water-saving irrigation techniques on these five crops is of utmost importance. This review delves into the significant role of various water-saving irrigation techniques in enhancing WP and crop yields and explores the benefits that water-saving irrigation technologies bring to modern agriculture. This will help producers to select the appropriate irrigation techniques in production practice to achieve better economic or ecological benefits, making this review a valuable source of theoretical reference for production practitioners and researchers.

## Sustainable solutions of embracing water constraints: unravelling water-saving irrigation techniques

### Typical water-saving irrigation technologies under the FI strategy: Drip irrigation technology for FI (DRFI)

**Fig. 1:**
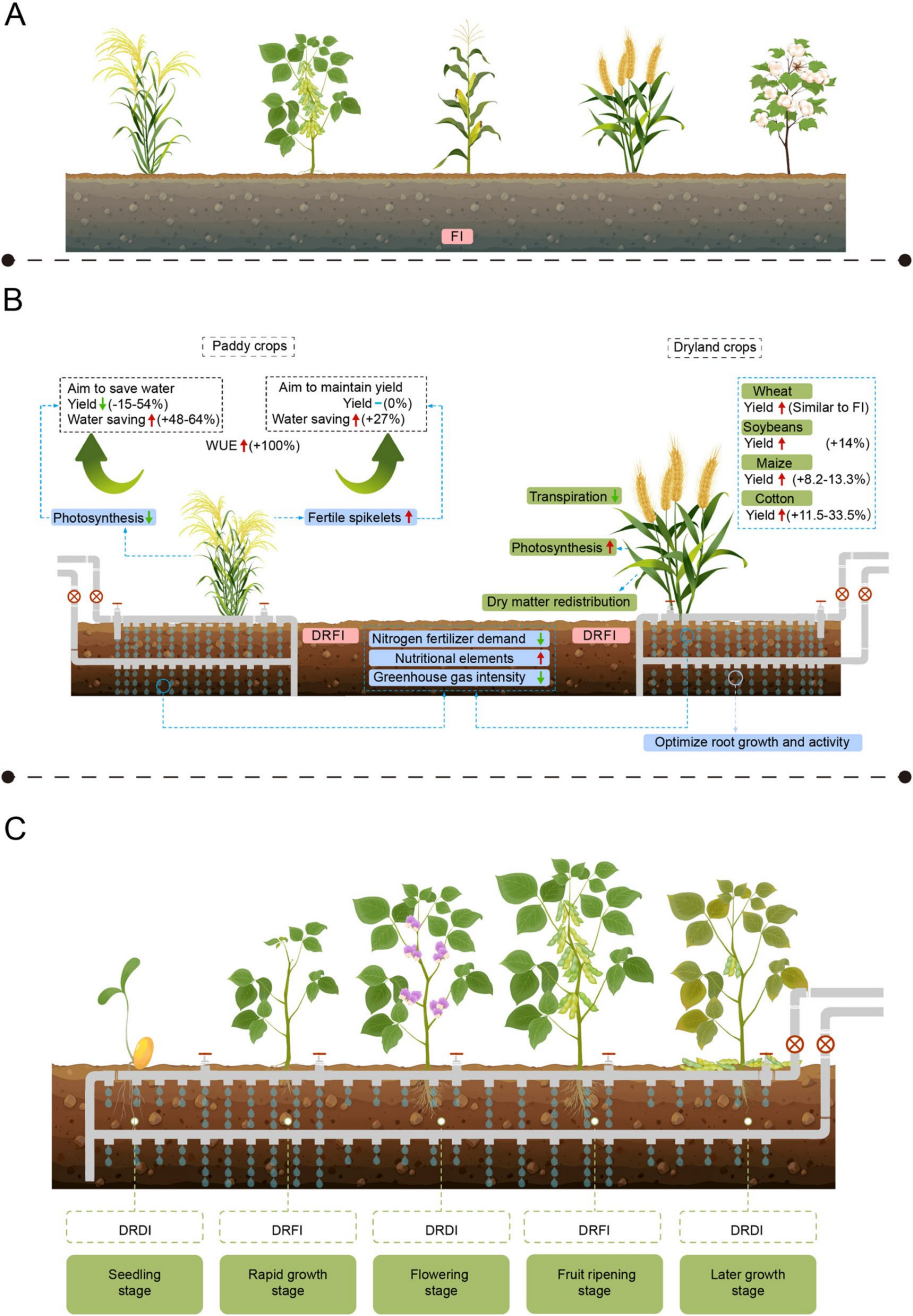
Utilization under drip irrigation and water saving irrigation. **A** Full irrigation (FI), strategy of irrigation by evapotranspiration with the amount of water required for high crop yield; **B** Impact of drip irrigation technology for full irrigation (DRFI) on crops. Where the red arrow represents an increase, the green arrow represents a decrease, and the blue rectangle represents an insignificant effect. **C** Optimal drip-reduced deficit irrigation (DRDI) use chart according to crop growth stage. Applying appropriate water deficit during the plant's seedling stage, flowering period, or later growth stage, and providing sufficient water during the rapid growth or fruit ripening stage seems to be a way to save water and ensure yield

FI is an irrigation approach that supplies crops with the precise amount of water required for optimal growth and high yields (Fig. 1A). Two primary quantitative benchmarks exist for FI. The first standard uses crop evapotranspiration (ETc), which calculates irrigation water demand by multiplying the reference crop evapotranspiration (ET0) with the crop coefficient (Kc), representing the crop's evapotranspiration (Hergert et al. 2016). The second standard employs field capacity as a reference, considering over 75% field capacity as indicative of FI (Haghighi et al. 2023; O'Shaughnessy et al. 2023). Various irrigation methods can achieve FI, among them, flood irrigation is the most traditional, but causes considerable water waste, while drip irrigation is one of the most typical water-saving forms of FI (Umair et al. 2019; Parthasarathi et al. 2018).

DRFI, short for drip irrigation technology for FI, involves the gradual and uniform release of water near plant roots through pipes or drippers, delivering water droplets to the soil surface (Bozkurt Çolak 2021; Liu et al. 2016a; Ma et al. 2022a; Wang et al. 2021b) (Fig. 1B). DRFI suits diverse crops, particularly those that demand ample water, are sensitive to water stress, or benefit from precise water and nutrient supply. However, it is unsuitable for submerged crops like rice (Bozkurt Çolak 2021; Parthasarathi et al. 2018). It can be adapted for varied soil types (Arbat et al. 2020), and also accommodates different climates. However, factors like farm size, layout, installation costs, maintenance needs, crop value, and potential water savings should be weighed for the economic viability of adopting DRFI (Sidhu et al. 2019).

DRFI presents an enticing solution for water conservation in rice cultivation. In the realm of rice, DRFI boasts double the WUE compared to traditional flood irrigation (Bozkurt Çolak 2021; Parthasarathi et al. 2018) (Fig. 1B). Research highlights that for water conservation priorities, DRFI can save 48–64% water while potentially causing yield reductions of 15–54%. The optimum balance lies in applying DRFI to save 49% water with only a 15% yield reduction (Bozkurt Çolak 2021; Sidhu et al. 2019; Fawibe et al. 2020; He et al. 2013; Wang et al. 2023). When preserving yield becomes the aim, DRFI can save about 27% water without compromising grain yield (Parthasarathi et al. 2018). Interestingly, some studies even propose that DRFI might augment yields by 15–23.5% while concurrently saving 23.3% water (Parthasarathi et al. 2023). In the context of dryland crops, DRFI emerges as a water-saving measure that boosts crop yield and WUE without the usual yield reductions (Wang et al. 2021c; Aydinsakir et al. 2021) (Fig. 1B). For instance, in wheat cultivation, DRFI curbs evapotranspiration by 11–26%, while matching flood irrigation in grain yield, yield composition, and aboveground biomass (Sidhu et al. 2019). In soybean cultivation, DRFI consistently elevates soybean yield and aboveground dry matter (AGDM) by an average of 14% (Chomsang et al. 2021). Similarly, in maize cultivation, DRFI has the potential to raise grain yield by 8.2–13.3% (sometimes even up to 119%) while enhancing WUE by 3.5–8.0% (Ma et al. 2022b; Gao et al. 2021). In cotton, DRFI increases average seed cotton yield by 11.5–33.5%, and saves about 30% of water (Rao et al. 2016; Liu et al. 2016a), significantly improving cotton yield and WUE (24.10–89.03%) (Wang et al. 2021b; Çetin and Kara 2019; Zong et al. 2021). In summary, for paddy crops like rice, DRFI offers substantial water savings and notable enhancements in WUE, albeit potentially accompanied by yield reductions. In the case of rainfed crops, DRFI can achieve noteworthy water savings, substantial improvements in WUE, and can maintain or even enhance crop yields.

DRFI's impact on yield varies based on crop type. Generally, except for rice, DRFI seldom leads to significant yield reductions. Well-planned DRFI strategies can even yield potential gains of about 10%. However, rice cultivation poses a unique situation where DRFI might entail yield reduction risks (Table 1). In rice fields, DRFI's yield influence is derived from reduced flag leaf photosynthesis during grain-filling and decreased post-flowering dry matter accumulation (Wang et al. 2023) (Fig. 1B). Conversely, higher yields result from increased fertile spikelets per panicle (Fawibe et al. 2020). Noteworthy studies show DRFI reshaping dry matter distribution and rice photosynthesis, promoting more fertile spikelets on primary branches and tillers, thus enhancing nutritional quality (Parthasarathi et al. 2018; Fawibe et al. 2020; Wang et al. 2023). Additionally, DRFI stimulates rice root growth, particularly in deeper soil layers, boosting robust grain development and yield (He et al. 2013; Parthasarathi et al. 2017). For dryland crops, DRFI can enhance plant height, net photosynthesis, chlorophyll (Chl) content, leaf area index (LAI), and leaf nitrogen (N) content while reducing transpiration (Umair et al. 2019; Zong et al. 2021; Guo et al. 2022) (Fig. 1B). Moreover, DRFI significantly impacts seed yield, mature aboveground dry matter (AGDM), and reproductive structures like soybean pods and cotton bolls (Wang et al. 2021c; Chomsang et al. 2021; Illés et al. 2022). DRFI also adeptly manages soil moisture and root growth (Parthasarathi et al. 2018; Ma et al. 2022a). In wheat, for instance, DRFI shapes root morphology, activity, and distribution of the active root system, prompts non-hydraulic root signals (nHRS) earlier, enhances yield, stability, and water productivity (Ma et al. 2022a). Similar outcomes are observed in cotton, where drip-irrigated cotton excels in water use efficiency at the 40 cm depth (Çetin and Kara 2019). DRFI augments soil water content (SWC) in the 0–60 cm soil layer, promoting fine root proliferation, enhancing water uptake, and boosting AGDM, lint, and seed cotton yield (Wang et al. 2021b). In summary, DRFI optimizes root growth and distribution, and influences crop yield by regulating photosynthesis and dry matter allocation (Table 2).

**Table 1:** Effect of different water-saving irrigation techniques on water use and yield

Technique	Species	Description	References
DRFI	Rice	Water saving: 48–64%, Yield: 15%—54% ↓, WUE: 2 times that of FI ↑	Bozkurt Çolak 2021; Parthasarathi et al. 2023; Wang et al. 2021c; Aydinsakir et al. 2021; Chomsang et al. 2021)
	Rice	Water saving: 27%, Yield: similar to FI	Wang et al. 2021b)
	Rice	Water saving: 23.3%, Yield: 15%—23.5% ↑	Ma et al. 2022b)
	Wheat	Water saving: 11%—26%, Yield: similar to FI, PIWUE: 36% ↑, CWP: 24.95% ↑, IWP: 19.59% ↑	Liu et al. 2016a)
	Wheat	Water saving: 42–53%	Parthasarathi et al. 2023)
	Soybean	Yield: 14%↑	Zong et al. 2021)
	Maize	Yield: 8.2%—13.3% ↑, WUE 3.5%—8.0% ↑	Parthasarathi et al. 2017; Guo et al. 2022)
	Cotton	Yield: Significantly increased ↑, WUE: 24.10–89.03% ↑	Fawibe et al. 2020; Illés et al. 2022; Zhu et al. 2013)
	Cotton	Water saving: 30%, Yield: 11.5%—33.5% ↑	Rao et al. 2016; Arbat et al. 2020)
TDI	Wheat	(50% TDI) Yield: 44% ↓; (75% TDI) WUE: 23%–28 ↑	Painagan and Ella 2022)
	Soybean	(50% TDI) Yield: 34.8% ↓; (20% TDI) Yield: 51.5% ↓	Yang et al. 2023)
	Maize	(60% TDI) Yield: 9.0—17.0% ↓	Khatun et al. 2021)
	Maize	(85%TDI) Yield: similar to FI	Pabuayon et al. 2019; Flynn et al. 2021)
	Cotton	(70–80% TDI) Yield: similar to FI	Wen et al. 2018)
DRDI	Wheat	Yield and WUE: significantly increases ↑	Ertek and Kara 2013)
(Compared to DRFI)	Soybean	(0% DRDI) Yield: 54%↓; (25% DRDI) Yield:32% ↓, (50% DRDI) Yield: 17% ↓; (75% DRDI) Yield: 8% ↓	O'Toole et al. 1980)
	Maize	(60% DRDI) Yield: 28%—45% ↓, WUE: 14% -15% ↑; (80% DRDI) Yield: 6%–9% ↓, WUE: 30%—42% ↑	Zhang et al. 2019)
	Maize	(75% DRDI) Yield: similar to DRFI	Eissa and Negim 2018)
	Maize	(70% DRDI) Yield: similar to DRFI; (40% DRDI) Yield: significant decrease, WUE: 21%↑	Himanshu et al. 2019)
	Maize	(70% DRDI) Yield: similar to DRFI, (40% DRDI) Yield: 25% ↓	Eltarabily et al. 2021)
	Cotton	(80% DRDI) Water saving: 17%, Yield: 6.4% ↓	Rao et al. 2016)
	Cotton	Yield: (50% DRDI) 13% ↓	Arbat et al. 2020)
	Cotton	WUE: 5.3% ↑	Cheng et al. 2021a)
PRDI	Wheat	Water saving: 11.6–17.3%, WUE: 17.2–20.3% ↑	Ke and Wan 2019)
	Soybean	(50% PRDI) Yield: 25—30% ↓, WUE: 53% ↑	Shi et al. 2014)
	Maize	Water saving: 34.4—36.8%, Yield: 6—11% ↓, WUE: significantly increased ↑	Cheng et al. 2021b)
	Maize	Yield: 4.62–20.71% ↑, WUE: 38.93% ↑	Mehrabi et al. 2021)
	Cotton	(70% PRDI) Yield: 4.44% ↓, WUE: 21% ↑	Karandish and Shahnazari 2016)
	Cotton	WUE: 21% -26% ↑	Kang et al. 1998; Wang et al. 2007)
	Cotton	(70% PRDI) Yield: 8% ↓	Tang et al. 2010)
AWD	Rice	Water saving: 25.7%, Yield: 5.4%% ↑, WUE: 24.2% ↑	Massey et al. 2014)
	Rice	Water saving: 28.8%%, Yield: 9.2–12.3% ↑	Sandhu et al. 2017)
	Rice	Water saving: 8%—41%, Yield: 0%—1% ↑, WUE: 11% -54%, ↑	Yang et al. 2004)

**Table 2:** Physiological and biochemical changes of crops under water-saving irrigation technology

Technique	Species	Description	References
DRFI	Rice	Photosynthetic efficiency decreased during grout period and dry matter accumulation decreased after anthesis	Chomsang et al. 2021)
	Rice	The number of fertile spikelets increased	Wang et al. 2021b; Wang et al. 2021c; Chomsang et al. 2021)
	Rice	Stimulates root growth, increases the number of deep roots and adenosine triphosphatase activity	Aydinsakir et al. 2021; Fan et al. 2022)
	Wheat; Maize; Soybean; Cotton	Increased plant height, net photosynthetic rate, chlorophyll content, chlorophyll fluorescence parameters, LAI and leaf N content, and reduced transpiration	Liu et al. 2016a; Zhu et al. 2013; Li et al. 2021)
	Soybean; Cotton	Influence on total seed yield, AGDM and number of branches, nodes and fruits	Gao et al. 2021; Zong et al. 2021; Felisberto et al. 2022)
	Wheat; Cotton	Regulates soil moisture and affects plant root growth	Wang et al. 2021b; Sidhu et al. 2019)
	Wheat	Optimize RMP, RAP, and MRA	Sidhu et al. 2019)
	Cotton	Increase the distribution of fine roots and enhance water absorption	Fawibe et al. 2020)
TDI	Wheat	Affects gas exchange and photosynthesis	Liu et al. 2013)
	Soybean	Proline content increased to maintain leaf water potential	Li et al. 2022)
	Maize	Reduce grain weight, density, crushing sensitivity, starch content, increase protein content, starch gelatinization temperature and free amino nitrogen	Comas et al. 2019)
	Cotton	Decrease in plant height, number of nodes and number of fruits	Wen et al. 2018)
	Maize; Cotton	Promote root downwards growth and reduce soil microbial content	Jones et al. 2009; Stamatiadis et al. 2016)
DRDI	Rice	Stimulate root growth and promote root depth	Tang et al. 2005)
	Wheat	Increase root length and root weight and aboveground biomass accumulation	Iqbal et al. 2021b)
	Soybean	Increased fat content and reduced protein content	O'Toole et al. 1980)
	Cotton	Reduced fiber strength and shortened fiber length	Arbat et al. 2020)
	Maize	LAI decreases, leaves curl, photosynthesis decreases and stomata closes	Eissa and Negim 2018; Singh et al. 2023)
	Maize	Seed number and dry leaf weight decreased, reduce grout rate and duration	El-Sadek 2014)
	Maize	Promote root growth, improve water absorption capacity	Eissa and Negim 2018; Himanshu et al. 2019)
PRDI	Wheat	Reduced LAI, leaf dry matter and leaf water content	Ke and Wan 2019; Liang et al. 2013)
	Wheat	ABA synthesis increased, while GA3 and IAA concentrations decreased	Liang et al. 2013; Graham-Acquaah et al. 2019)
	Wheat	Influence hydraulic conductivity	Batool et al. 2019a)
	Maize; Soybean; Wheat	Higher proline, soluble sugars and protein content; lower superoxide dismutase, peroxidase and APX activity	Ke and Wan 2019; Liang et al. 2013; Zhang et al. 2023a)
	Maize	Enhance root penetration; Influence root distribution	Hu et al. 2011; Iqbal et al. 2022)
	Maize; Wheat	Pore closure and increased resistance to water diffusion	Cheng et al. 2021b; Graham-Acquaah et al. 2019)
	Cotton	Increase ABA content in leaves	Wang et al. 2007)
	Cotton	Low GS and smaller LAI	Tang et al. 2010)
AWD	Rice	Increased aroma biosynthesis and proline content	Chaurasiya et al. 2022; Qi et al. 2023)
	Rice	Leaf development was affected and photosynthesis was enhanced after water application	Chaurasiya et al. 2022; Sandhu et al. 2017)
	Rice	Increased ABA and isopentenyladenine concentration, and decreased leaf trans-zeatin concentration	Deng et al. 2021)
	Rice	Stimulates root growth and delays root senescence	Sandhu et al. 2017)
	Rice	RAP and root-to-crown ratio improved	Martínez-Eixarch et al. 2021)
	Rice	Slightly higher chalkiness, lower setback viscosity	Song et al. 2021b)
	Rice	Increased abscisic acid content in the seeds	Wu et al. 2021)
	Rice	Increasing amino acids and phenolic acids and reducing lipids and alkaloids	Zhang et al. 2023b)
	Rice	Soluble sugar, protein accumulation, proline, antioxidant enzymes and glutathione contents were affected	Chaurasiya et al. 2022)
	Rice	Increased the reactivation of non-structural carbohydrates and increased the activity of sucrose—related enzymes and peroxidase	Wu et al. 2021)

In paddy fields and water-cultivated crops, DRFI reduces methane emissions by 78% (Parthasarathi et al. 2023), trims nitrogen fertilizer demand by 20% while maintaining yield, enhances N use efficiency (NUE) (Sidhu et al. 2019). Under DRFI, rice rhizosphere soil shows lower NH_4_-N, available potassium (K), and exchangeable manganese (Mn), but higher NO_3_-N and zinc (Zn) (Zhu et al. 2013). In rain-fed crops, DRFI raises surface soil water, safeguards groundwater, and affects water and nitrogen distribution (Parthasarathi et al. 2018; Ma et al. 2022a; Çetin and Kara 2019; Fan et al. 2022). DRFI boosts nitrogen, phosphorus, and potassium accumulation, reduces nitrogen fertilizer demand by 20%, maintains yield, and enhances nutrient use efficiency (Sidhu et al. 2019; Wang et al. 2021c). Combining DRFI with fertilizers like urea and ammonium sulfate improves WUE and NUE (Li et al. 2021). DRFI lowers greenhouse gas (GHG) intensity (12–27%), aids global warming mitigation, and supports sustainable agriculture (Gao et al. 2021). Overall, DRFI enhances plant nutrient use, soil water and nutrient efficiency, and cuts GHG emissions (Table 3).

**Table 3:** Effect of different water-saving irrigation on plant uptake of soil elements or environment

Technique	Description	References
DRFI	Methane emission fluxes down 78%	Ma et al. 2022b)
	Promote the accumulation of N salt and the mobilization of P	Liu et al. 2016b)
	Increased NO 3-, K, Zn and reducible Mn concentrations	Hanif et al. 2020)
	Higher plant N, P, K accumulation and use efficiency, 20% reduction in nitrogen fertilizer requirement	Parthasarathi et al. 2023; Gao et al. 2021)
	Combining with fertilizers can further increase NUE	Allakonon et al. 2022)
	Reduced GHG intensity (12–27%)	Guo et al. 2022)
TDI	NUE increased with the increase of irrigation	Painagan and Ella 2022)
	Decreased water supply leads to 50% reduction in nutrient N	Aydinsakir 2018)
	40–60 cm SOC with N concentration decreases and aggregate stability decreases with increased irrigation	Stamatiadis et al. 2016)
DRDI	Reduced plant uptake of N, P, and K	Gao et al. 2021; Zhang et al. 2019)
	Reduces the total plant uptake of N, P, and K	Zhang et al. 2019)
	Seed stage leads to 50% reduction in nutrient nitrogen	Aydinsakir 2018)
PRDI	Increased root N uptake with a compensatory effect on N uptake	Batool et al. 2019b)
	Increased plant N uptake, WUE and NUE	Xia et al. 2021)
	Total fertilizer and PRDI are effective modes for plants grow	Fu et al. 2017)
	Promote leaf nitrogen accumulation	Wang et al. 2012)
	Increase leaf N content	Hu et al. 2011; Liang et al. 2013)
AWD	Altering soil pH and accelerating soil nutrient cycling and transfer	Sandhu et al. 2017)
	Promote nutrient uptake, distribution and transfer	evidence from Bangladesh et al. 2022)
	Improve phosphorus transport and metabolic efficiency	Wu et al. 2021)
	Combined application of nitrogen can reduce nitrogen leaching	Liao et al. 2020)
	Improving the efficiency of N, P and K utilization	Wang et al. 2022; Wu et al. 2021)
	Decreased content of S, Ca, Fe, and As; increased content of Mn, Cu, and Cd	Deng et al. 2021)
	Reduce As concentrations and increase Cd, Cu, Se and Zn	Ullah et al. 2017)
	Pb is accumulated in the roots; As and Cd are transported to the above-ground parts and accumulated in the seeds	Lopez et al. 2020; Gregorio Jorge et al. 2020)
	Combined with P application can further reduce the concentration of heavy elements such as Pb and As in grains	Norton et al. 2017; Lopez et al. 2020)
	Plants with reduced tolerance to Na	Massey et al. 2014; evidence from Bangladesh et al. 2022)
	Reduce GHG emissions and water use	Massey et al. 2014; Ullah et al. 2017; Du et al. 2018)
	Reduces methane emissions by 30–70%	Cutler et al. 2010)
	87.1% reduction in cumulative CH_4_ emissions	Song et al. 2020)
	Reduction of annual methane emissions (-51%)	Xue et al. 2008)
	Increases N_2_O emissions by 28.8%, reducing GWP by 62.9%	Song et al. 2021a)
	Reduce GWP by 90%	Ullah et al. 2017)

#### Traditional deficit irrigation techniques (TDI) based on directly reducing water usage

**Fig. 2:**
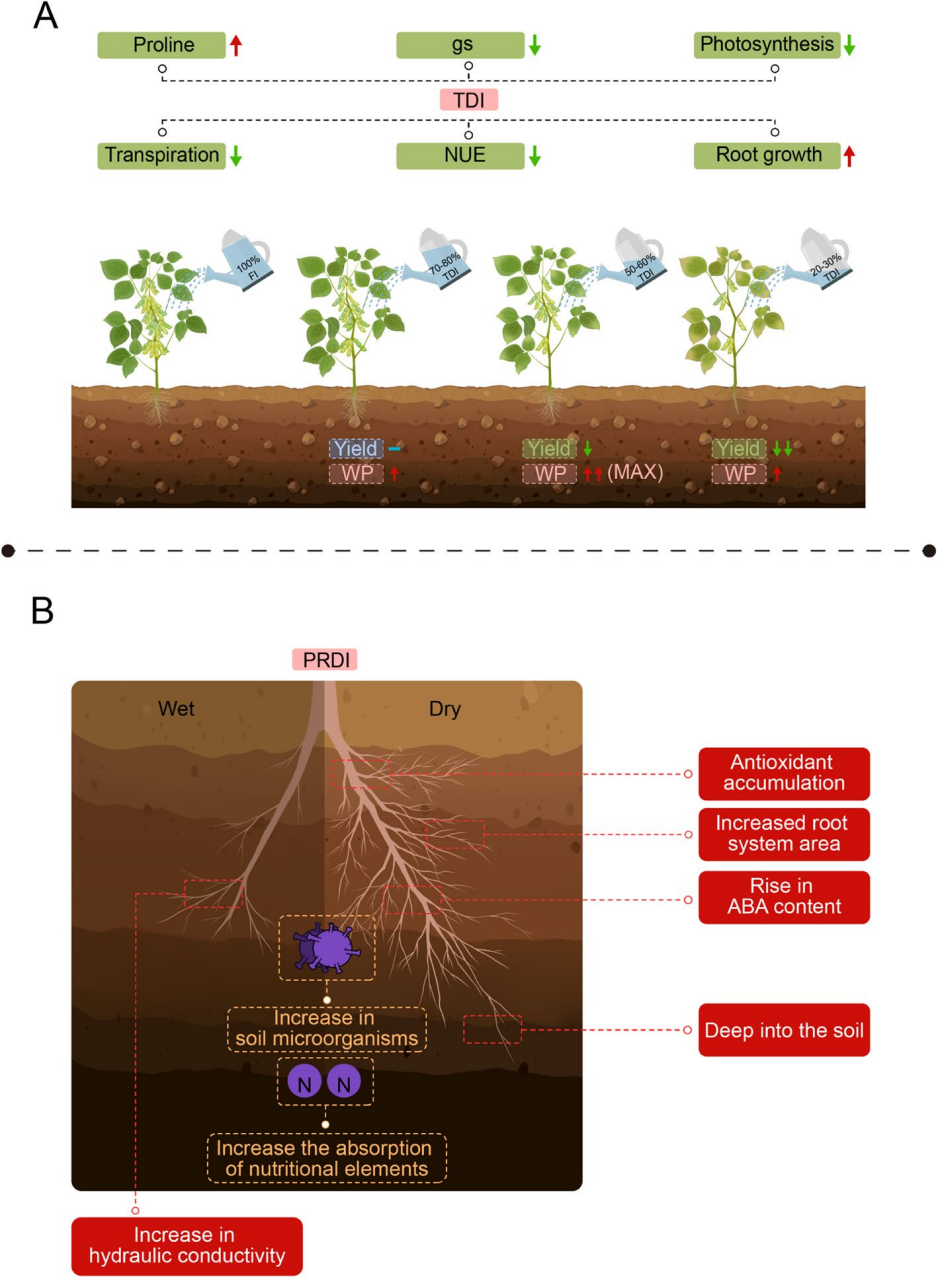
Effects of traditional deficit irrigation technique (TDI) and partial root zone drying-based deficit irrigation techniques (PRDI) on crops. **A** Both TDI can improve WP of crops, but as irrigation water decreases, crop growth and development are affected and yields gradually decrease. The left half of the picture represents the wet root zone, which has increased hydraulic conductivity, and the right half of the picture represents the dry root zone, which has a larger area and explores deeper soil, along with increased ABA and antioxidant content. In addition, under PRDI, plants can better absorb nutrients and have higher soil microbial content

Traditional deficit irrigation techniques (TDI), do not use other forms of irrigation, such as drip or sprinkler irrigation, and can save a significant amount of irrigation water (García-Tejera et al. 2018; Peake et al. 2016) (Fig. 2A). TDI suits regions with limited water resources or a focus on conservation, aiming for water efficiency and acceptable yields (Peake et al. 2016). It can be applied across multiple crops, but requires knowledge of the water needs and stress tolerance at all growth stages (Felisberto et al. 2022; Hanif et al. 2020). Soil characteristics influence TDI suitability, with good water-holding capacity and root permeability favouring deficit irrigation (Naghdyzadegan Jahromi et al. 2022). Climate, resources, evapotranspiration, and rainfall must be weighed for TDI feasibility (Allakonon et al. 2022). Cost–benefit analysis must also be conducted to determine if the reduced water input aligns with the economic goals of agricultural operations.

However, TDI leads to reduced production (Fig. 2A). Studies reveal that 50% TDI results in yields that are about 44% of FI in wheat (Naghdyzadegan Jahromi et al. 2022), 6% and 60% TDI decreases corn yields by 9.0%-17.0% compared to FI (Li et al. 2022). Similarly, soybean yields decreased by 34.8% and 51.5% with 50% and 20% TDI, respectively (Amani Machiani et al. 2021). Nonetheless, TDI enhances plant WP (Peake et al. 2016). Studies show that reducing irrigation by 15% maintains yields similar to those of FI, and 60% TDI achieves peak WP (Painagan and Ella 2022; Himanshu et al. 2023). In wheat, 75% TDI enhances WUE by about 23%-28% (Naghdyzadegan Jahromi et al. 2022). For cotton, a 70%-80% deficit maintains lint yield comparable to FI (Wen et al. 2018). Thus, TDI balances WUE and yield, avoiding strict yield maximization. TDI significantly improves WP, and its yield impact primarily hinges on water consumption, where a 40%-50% or greater water shortage notably reduces yield for most crops, with magnitude increasing as water supply diminishes (Table 1).

TDI impacts crop quality and yield through various mechanisms (Fig. 2A) and triggers a range of physiological changes in plants (Table 2). Initially, the plant's water-deficit tolerance mechanism is inadequate (Felisberto et al. 2022; Khatun et al. 2021). For instance, soybeans initially increase proline production to maintain acceptable leaf water content and potential under water stress, but as TDI progresses, this mechanism becomes insufficient, resulting in severe yield losses (Felisberto et al. 2022). Secondly, water deficit disrupts several plant physiological processes. In wheat, it affects the net photosynthetic rate, stomatal conductance (Gs), transpiration efficiency, and intrinsic water use efficiency, which together limit photosynthetic productivity and yield due to soil water deficit (Yang et al. 2023). In cotton, drought stress influences plant height, knot number, seed number, and yield (Wen et al. 2018). TDI also alters root growth and soil microbial communities. Moderate to mild stress treatments in cotton promote downward root growth, extracting water from deeper soil profiles (60–100 cm) in late season. In late-stage maize growth under TDI, there is enhanced root growth in the 40–60 cm soil layer and decreased soil microbial biomass (Pabuayon et al. 2019; Flynn et al. 2021). TDI's impact on crop WUE and irrigation WUE also depends on the growth stage during which it is applied. For cotton, growth is divided into four stages: first leaf to first square (GS1), flower initiation/early bloom (GS2), peak bloom (GS3), and cut out, late bloom, and boll opening stage (GS4). GS2 and GS3 are the most sensitive to water stress, while GS4 is least sensitive (Himanshu et al. 2023). For maize, TDI at any stage lowers dry matter and grain yield, with single-stage TDI impact is only on grain yield, whereas TDI spanning multiple stages impacts both biomass and grain yield (Igbadun et al. 2008). Maize varieties exhibit improved WP under TDI, favouring early-season TDI for yield (Allakonon et al. 2022). For cottonseed, in normal years, the strategy of replacing 90% ETc in GS1 to GS3 and 30% ETc in GS4 is optimal for higher yield (Himanshu et al. 2023).

TDI influences plant physical traits, chemical composition, and other attributes. For instance, maize experiences reduced kernel weight, density, and breakage susceptibility as irrigation levels decrease (Liu et al. 2013). Under low irrigation, maize starch content decreases by around 0.9% (Liu et al. 2013; Jones et al. 2009). Simultaneously, decreasing irrigation elevates starch gelatinization temperature and free amino N, while ethanol production diminishes by about 4.0% (Liu et al. 2013). Therefore, TDI can cause plants to respond to drought stress and maintain their growth through a series of physiological and chemical reactions.

TDI application may reduce plant NUE. Studies indicate that wheat NUE increased by 2.8%, 6.9%, and 7.8% for 50%, 75%, and 100% TDI, respectively, compared to the initial and subsequent years with 0% TDI (Naghdyzadegan Jahromi et al. 2022). TDI can lead to a 50% N nutrition decrease due to limited water supply (Stamatiadis et al. 2016). Under TDI conditions, soil organic carbon (SOC) concentration in the 0–20 cm depth diminishes with higher total irrigation, with SOC and N concentrations in the 40–60 cm depth also declining significantly. Additionally, aggregate stability in the 0–20 cm depth generally decreases with increased irrigation (Flynn et al. 2021). Therefore, supplementation with N fertilization is recommended when applying TDI to meet the growth and production needs of plants (Table 3).

#### Drip irrigation-based deficit irrigation techniques

Drip-reduced deficit irrigation (DRDI) curtails irrigation volume via DRFI, creating water deficit (Fig. 1C). DRDI safeguards groundwater and 100% DRFI combined with moderate DRDI yields similar net benefits as FI, preserving crop yield and farm income (Fan et al. 2022). DRDI aligns with DRFI's applicability and is suited to most crops, except aquatic plants. It is especially fitting for water-scarce regions or water conservation is required (Zhao et al. 2022; Comas et al. 2019). Versatile across crops and soil types, DRDI strategically applies water during critical growth stages for water efficiency, maintaining acceptable yields (Wan et al. 2022).

In soybean, compared to 100% DRFI, yield reductions of 54%, 32%, 17%, and 8% were observed with 0%, 25%, 50%, and 75% DRDI, respectively (Aydinsakir 2018). In cotton, 80% DRDI conserved 17% water but only decreased yield by 6.4% (Rao et al. 2016). Additionally, 50% DRDI lowered seed cotton yield by 13% (Liu et al. 2016a). Seed cotton and lint yield rose with increased DRDI water application (Wang et al. 2021c). Yet, in maize, with 60% and 80% DRDI, grain yield was reduced by 28%-45% and 6%-9%, straw yield by 40%-66% and 14%-20%, respectively (Eissa and Negim 2018). DRDI influenced maize ear weight, grain weight per ear, and seed index among other properties. For instance, with 60% DRDI, ear weight, grain weight per ear, seed index shrank by 20%, 13%-23%, and 16%-25% versus 100% DRFI, while with 80% DRDI, they were reduced by 4%-5%, 2%-3%, and 8%-10%, respectively. Additionally, biological yield with 60% DRDI decreased by 47%-58% (Eissa and Negim 2018). However, DRDI does not always reduce maize biomass and grain yield, with 75% DRDI causing no yield reduction (Zhao et al. 2022). In a maize DRDI study, 70% DRDI maintained yields near 100% DRFI for two years, while 40% DRDI led to a notable reduction in yield (Singh et al. 2023). Similar findings have been observed in other studies. 70% DRDI had the lowest impact on sweet maize yield, with higher quality and no reduction in yield, while 40% DRDI resulted in the lowest fresh ear yield, an approximate 25% reduction, but with the highest protein and sugar content (Ertek and Kara 2013). As such, 70–80% DRDI does not notably affect yield, similar to DRFI, while excessive water deficit significantly reduces yield. Additionally, DRDI significantly reduced fibre strength, fibre length in cotton (Liu et al. 2016a). In soybeans, DRDI-induced water deficit raised oil content and lowered protein content (Aydinsakir 2018), influencing crop quality. Overall, DRDI can enhance WP from DRFI. Yield considers water usage, crop type, with trends akin to TDI vs FI. Severe water deficit magnifies yield reduction. Yet, DRDI's yield impact varies by crop. For soybeans and cotton, ~ 20% water deficit slightly reduces yield, but maize is unaffected (Table 1). Generally, DRDI combines the benefits of DRFI and TDI.

Utilizing DRDI maximizes water conservation while maintaining crop yields. Research indicates that applying DI throughout maize's growth stages, except for the later vegetative ones, achieves yields akin to DRFI, curtailing ETc by 15–17% (Comas et al. 2019). In the late nutritional stage, water deficit leads to a slight reduction in LAI, significant leaf curling, and lowered photosystem II efficiency (quantum yield), however, recovery occurs upon rewatering (Comas et al. 2019; O'Toole et al. 1980). Favourable light conditions and photosystem II's resilience post-stress suggest biomass and yield reduction are due to temporary stomatal closure, decrease in photosynthesis, or fleeting wood conductivity loss—all of which are remedied by water replenishment (Zhao et al. 2022; Comas et al. 2019). A parallel study, revealed that water deficit during maize's nutritional stage curbs grain count, dry leaf weight, impeding the potential grain filling rate, while during mature growth, it directly truncates grain filling rate and duration, chiefly impacting yield (Zhang et al. 2019). A similar pattern emerges in cotton, where water deficit during initial or final growth stages barely affects seed cotton yield, but during peak flowering, it markedly reduces yield (Himanshu et al. 2019). Thus, judicious water deficit application during seedling, flowering, or later growth, paired with adequate water during rapid expansion or fruit maturation, conserves water and ensures yield (Cheng et al. 2021a) (Fig. 1C).

DRDI concurrently triggers physiological shifts in crops (Table 2). Moderate irrigation reduction diminishes soil moisture status while enhancing dry matter accumulation in preharvest organs, which is later distributed to grains during filling (Wan et al. 2022). It also bolsters plants' soil moisture extraction capabilities, with root system enhancement pivotal for crop adaptation to water scarcity (Zhao et al. 2022; Singh et al. 2023). Compared to 100% DRFI, 70% DRDI raised root length density (RLD), and both 70% and 40% DRDI boosted soil water consumption (Singh et al. 2023). In rice, elevated drought levels lead to reduced SWC, reducing root water uptake, prompting root growth, deepening root penetration, and elevating WUE. However, this increased WUE was accompanied diminished water availability, culminating in lower rice and grain yield, and growth traits like plant height, tiller count, panicle length, panicle weight, and grain count per panicle showing a decline (Eltarabily et al. 2021). Furthermore, upping DRDI frequency could augment root length, root weight, and aboveground biomass accumulation in wheat (Chen et al. 2021a).

DRDI influences plant WUE and N, P, and K use efficiency. DRDI yields higher WUE than DRFI. Moderate irrigation reduction significantly enhances wheat WUE and returns (Wan et al. 2022). Cotton's overall WUE improves by about 5.3% under DRDI (Cheng et al. 2021a). Maize's WUE under 75% DRDI surpasses 100% DRFI (Zhao et al. 2022) and 70% DRDI elevated WUE by 21% compared to 100% DRFI (Singh et al. 2023). Meanwhile, two maize growth seasons that experienced 60% DRDI saw WUE increase by 14% and 15%, while 80% DRDI elevated it by 30% and 42% (Eissa and Negim 2018). However, DRDI-induced water stress can lower N, P, and K uptake by plants (Parthasarathi et al. 2023; Eissa and Negim 2018). For maize, a 60% DRDI reduces total N, P, and K uptake by 21%, 25%, and 21%, while an 80% DRDI reduces it by 5%, 10%, and 13%, respectively (Eissa and Negim 2018). Water scarcity during the seed stage alters N distribution, causing a 50% decrease in nutrient N (Stamatiadis et al. 2016). In summary, DRDI is a more water-efficient irrigation method than DRFI, but water reduction can affect N, P, and K absorption by plants (Table 3).

#### Partial root zone drying-based deficit irrigation techniques (PRDI)

PRDI divides the root zone into irrigated and dry halves (El-Sadek 2014; Iqbal et al. 2021a; Tang et al. 2005), inducing wet and dry areas that trigger water stress-related physiological responses (El-Sadek 2014; Iqbal et al. 2021a; Tang et al. 2005; Iqbal et al. 2021b). By generating chemical signals in the dry roots, PRDI significantly reduces water use and improves WUE (El-Sadek 2014; Iqbal et al. 2021a; Tang et al. 2005). PRDI suits diverse soil textures (except sandy soils) for most plants (Lekakis et al. 2011). PRDI, which is applicable to crops facing drought stress, benefits from effective water distribution and permeable roots. While PRDI is adaptable to many climates, it demands vigilant monitoring and adjustments to lessen risks (Iqbal et al. 2021a; Iqbal et al. 2021b; Kang et al. 1998; Tang et al. 2010; Barideh et al. 2018).

PRDI, an efficient water-saving irrigation method, enhances WUE, root development and distribution (Fig. 2B), and biomass production (Iqbal et al. 2021a; Iqbal et al. 2021b; Kang et al. 1998; Tang et al. 2010). On average, PRDI boosts WUE by 82% (Sadras 2008). At 50% PRDI, soybeans biomass might decreased by 25–30%, yet half of the irrigation water is conserved, elevating WUE by about 53% (Wakrim et al. 2005). Some studies find no significant crop yield difference (maize, wheat, cotton) with alternate partial rootzone irrigation (Cheng et al. 2021b). For instance, maize roots alternately exposed to dry, 55%, or 65% field capacity soil reduced water intake by 34.4–36.8%. Despite a mere 6–11% reduction in total biomass and yield as compared to well-irrigated plants, WUE surged (Kang et al. 1998). Moreover, PRDI conserved 15.2% maize season water, increased yield by 4.62–20.71%, and elevated WUE by 38.93% (Karandish and Shahnazari 2016; Al-Kayssi 2023). In cotton, 30% irrigation reduction under PRDI led to only 4.44% yield loss, which was statistically insignificant, with earlier flowering, quicker harvesting, and higher economic returns (Tang et al. 2010). Cotton's PRDI WUE outpaced that of FI and TDI by 21% and 26%, yielding greater biomass and WUE (Iqbal et al. 2021a; Li et al. 2017a). Some studies show PRDI saving 30% water, yet still yielding as many cotton bolls as FI, and an ~ 92% total lint yield (Tang et al. 2005). For wheat, PRDI decreased total water consumption per plant by 11.6–17.3%, and markedly increased WUE by 17.2–20.3% (Shi et al. 2014). Overall, PRDI significantly bolsters WP. While limited in yield potential compared to TDI, PRDI mitigates water deficit-induced yield decline with most crops experiencing only a minor decrease in yield (30%-40% water deficit) (Table 1).

PRDI treatment enhances root development, which is pivotal for plant material metabolism and information exchange (Table 2). Roots detect and positively respond to decreasing soil moisture (Caixia et al. 2015). Initially, PRDI encourages maize roots to penetrate soil more vigorously (Karandish and Shahnazari 2016; Caixia et al. 2015). Spatially, PRDI significantly influences maize's RLD distribution. Maximum RLD occurs in 10–20 cm soil, decreasing with depth. Water uptake by maize roots under PRDI relies on soil moisture and RLD distribution (Caixia et al. 2015). During nutritional growth, root water uptake efficiency (RWA) predominantly arises from the 10–30 cm soil layer, while during reproductive growth, the 20–70 cm soil layer's root system dominates water uptake (Caixia et al. 2015; Hu et al. 2011). After the filling stage begins, the surface soil root system gradually ages, and the deep soil RLD increases slightly (Caixia et al. 2015; Hu et al. 2011). Therefore, the renewal of soil water below 70 cm benefits the filling of maize (Caixia et al. 2015). PRDI also shapes maize's soil-root hydraulic conductivity, as soil water absorption hinges on root growth and distribution (Mehrabi et al. 2021), where crop water use relates significantly to hydraulic conductivity in the irrigated root zone. PRDI markedly compensates RWA, boosting total hydraulic conductivity in the irrigated root zone by 49.0–92.0% over FI (Hu et al. 2011). Furthermore, PRDI's non-irrigated root zone has higher hydraulic conductivity than a completely non-irrigated zone, contributing to total crop water uptake even without irrigation, a factor in PRDI's elevated WUE (Hu et al. 2011). PRDI enhances soil ventilation, increasing soil microbial abundance in the root zone, with mild soil water shortage increasing soil microbial abundance in PRDI (Wang et al. 2007). PRDI maintains a higher microorganism count at similar soil moisture versus FI, promoting plant-microorganism coexistence and fostering ecological balance (Wang et al. 2007; Ke and Wan 2019).

Leaf Chl content links positively to plant drought tolerance, and leaf water potential assesses water-holding capacity, while Gs evaluates plant response to low water conditions (Kang et al. 1998; Tang et al. 2010; Iqbal et al. 2022). Compared to FI, PRDI curbs LAI, leaf dry matter, and leaf relative water content (Shi et al. 2014; Liu et al. 2022). PRDI also elevates abscisic acid (ABA), a signaling chemical for direct/indirect stomatal behavior regulation, in leaves (Li et al. 2017a). While PRDI induces drought stress in root zones, it notably bolsters stomatal resistance to water diffusion, promoting more closure (Kang et al. 1998; Batool et al. 2019a), reducing transpiration by narrowing the stomatal opening (Tang et al. 2010). Although transpiration rates drop significantly, maize's photosynthetic rate and leaf water content show no significant change under PRDI (Kang et al. 1998; Shi et al. 2014). In wheat, the leaf photosynthesis rate did not significantly drop within two PRDI years, but Gs was reduced by 12% and 7% (Mehrabi and Sepaskhah 2019). Similar behaviors are observed in cotton, with PRDI causing lower Gs and smaller LAI than FI (Tang et al. 2005).

PRDI mitigates the impact of water stress through the regulation of physiological parameters (Xiancan Zhu and Liu 2018). PRDI results in more dry matter accumulation than TDI, lowered malondialdehyde, and higher proline, soluble sugars, and proteins in maize, soybean, and wheat. PRDI concurrently curbs superoxide dismutase (SOD), peroxidase (POD), and ascorbate peroxidase (APX) activities in maize and soybean, aiding water stress coping (Shi et al. 2014; Liu et al. 2022; Xiancan Zhu and Liu 2018). Under PRDI cotton accumulates osmolytes (sugar, proline) and antioxidants (SOD, POD, catalase (CAT), APX), raising SOD, POD, CAT, and APX by 19.86%, 43.90%, 20.51%, and 21.11%, respectively (Iqbal et al. 2022). Partial root zone drought stress in wheat intensifies partial root zone stress, sparking the operation of nHRS (Liu et al. 2022; Batool et al. 2019a). nHRS-mediated signal transduction ups ABA production, prompts the generation of reactive oxygen species (ROS, curbs cytokinin synthesis, amplifying plant antioxidant defences and proline content (Batool et al. 2019a; Batool et al. 2019b).

Compared to FI, PRDI heightens N and water utilization efficiency in plants (Table 3). PRDI notably enhances N absorption in the irrigated root zone, with a marked compensatory N uptake effect (Hu et al. 2009). Within five days post-irrigation, the root zone rapidly regains high N inflow (Hu et al. 2009; Xia et al. 2021). Additionally, PRDI elevates wheat's RLD and root mass density by 48% and 32%, respectively, within two years after nitrogen fertilization (Mehrabi et al. 2021). Enhanced N utilization in the root system under PRDI propels crop root development. Some reports suggest PRDI bolsters maize's N absorption, WUE, and NUE with 0.18 g N per kg, leveraging plant-soil N synergy (Fu et al. 2017). Thus, applying total fertilizer as basal in conjunction with PRDI has emerged as an effective water and fertilizer strategy (Liang et al. 2013). Compared to TDI and FI, PRDI plants exhibit superior root biomass and root-to-shoot ratio. Higher N application significantly boosts leaf N accumulation, with PRDI plants amassing more N in leaves than their TDI counterparts (Wang et al. 2012).

#### Crop-specific alternate wetting and drying (AWD) deficit irrigation techniques

**Fig. 3:**
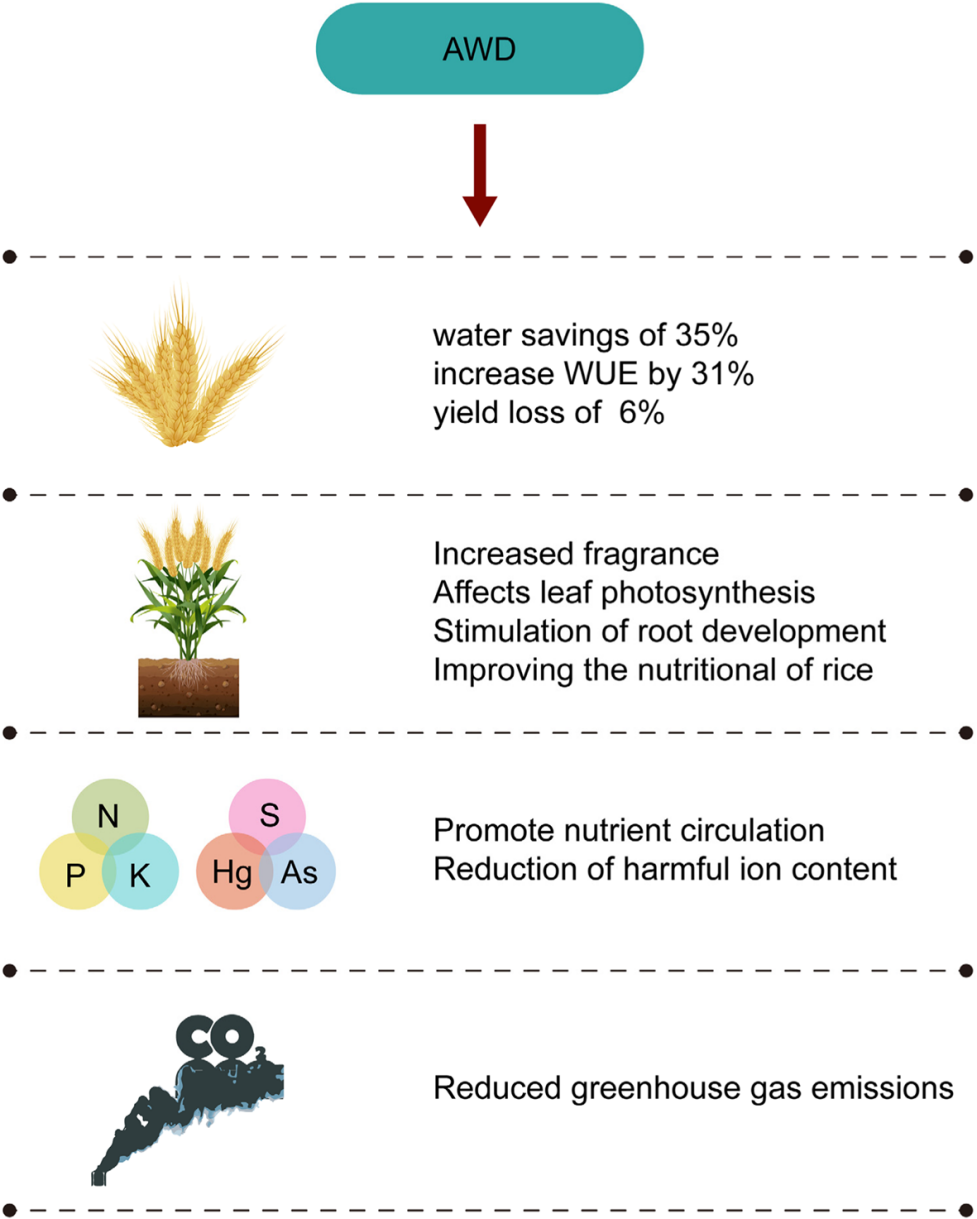
Impact of alternate wetting and drying (AWD) on rice. The upper part of the graph represents that AWD saves water while significantly increasing WUE and has no significant effect on yield; the lower part of the graph represents that AWD promotes plant growth; the left part of the graph represents that AWD promotes nutrient uptake and reduces harmful ions; the right part of the graph represents that AWD reduces greenhouse gas emissions

AWD introduces unsaturated soil conditions during the growth season, intermittently halting irrigation to allow water to recede until the soil reaches a specified moisture content before reflooding occurs (Carrijo et al. 2017; Graham-Acquaah et al. 2019; Ashraf et al. 2022). Two main methods quantify the AWD threshold: field water level (FWL) and soil water potential (SWP) (Carrijo et al. 2017). Typically, AWD's threshold is FWL reaching 15–18.5 cm below the soil or SWP between -15 kPa and -20 kPa (Carrijo et al. 2017; Zhang et al. 2023a). Rice is sensitive to unsaturated soil (Bouman and Tuong 2001) and AWD has been established in rice practices, especially in water-scarce areas, enhancing agricultural production (Fig. 3). Primarily for paddy or flooded rice, AWD suits clayey or loamy soils with good water retention (Carrijo et al. 2017; Zhang et al. 2023a).

AWD in rice cultivation can save up to 35% of water, increase WUE by 31%, with a yield loss of only around 6%, as confirmed by multiple studies (Carrijo et al. 2017; Graham-Acquaah et al. 2019; Zhang et al. 2023a; Massey et al. 2014; Song et al. 2021a; Song et al. 2021b). AWD duration has no yield impact during the vegetative and reproductive stages (Carrijo et al. 2017). Compared to FI, AWD reduces water use by 25.7% on average, with 23.4% water savings while maintaining yield (Carrijo et al. 2017; Chaurasiya et al. 2022). AWD's water-saving effect surpasses yield reduction, leading to 24.2% higher WP than FI (Carrijo et al. 2017). Additionally, AWD cuts rice irrigation by up to 28.8%, enhancing WUE (Haque et al. 2022), and potentially increasing yield by 9.2–12.3% (Li et al. 2018). In clay paddies, optimal AWD reduces water by 8%-41%, raising WP by 11%-54%, and yield by 0%-1% (Cheng et al. 2022). Furthermore, AWD yields larger grains, up 12.0–15.4% (Norton et al. 2017). Lower percolation and leakage contribute to decreased water usage in AWD (Carrijo et al. 2017). Increased filled grains, heavier inferior grains, and more tillers enhance yield under AWD (Li et al. 2018; Norton et al. 2017). Reports show AWD improves WP significantly, with varying yield effects (Table 1). AWD's impact depends on implementation conditions, as arid regions or severe deficits may affect yield. AWD timing also matters, especially during sensitive water periods, impacting yield, with careful planning having the potential to increase yield to some extent.

AWD triggers diverse physiological changes in rice (Table 2). Research has demonstrated that AWD treatment during both vegetative and reproductive stages elevates endogenous proline levels, a precursor for creating grain aroma compounds such as 2-acetyl-1-pyrroline (2-AP), thereby amplifying rice aroma through increased 2-AP production by 5%-15% (Ashraf et al. 2022; Zhang et al. 2023b). Leaf development is affected by AWD, showing slight elevation in photosynthetic rates (Ashraf et al. 2022; Li et al. 2018). Nevertheless, AWD-influenced leaves exhibit higher ABA content and a 37% increase in isopentenyladenine, while trans-zeatin decreased by 36% (Norton et al. 2017). Thirdly, wetting and drying cycles promote rhizosphere ecology, delaying root senescence, and stimulating growth (Li et al. 2018). AWD enhances root activity pattern and root-to-shoot ratio, vital for absorption capacity and reduced transpiration (Zhang et al. 2017). The rise in CO_2_ concentration under AWD bolsters root growth via increased root ABA synthesis (Wang et al. 2022). Grain quality is also impacted by AWD, with treated rice showing minorly elevated chalkiness and reduced setback viscosity (Graham-Acquaah et al. 2019). AWD enhances polished rice's nutritional profile by increasing amino acid and phenolic acid content, while lowering lipids and alkaloids (Song et al. 2021b). AWD variably affects soluble sugars, proline, proteins, and enzyme activity, with higher malondialdehyde and H_2_O_2_ levels than FI (Ashraf et al. 2022).

Moisture fluctuations during AWD enhance nutrient cycling, microbial dynamics, and nutrient mineralization by disrupting soil aggregates and triggering physical and biological changes, optimizing the resource utilization efficiency for benefiting plants (Li et al. 2018; Sandhu et al. 2017). The AWD system cyclically alters soil pH, accelerating nutrient circulation and transfer in the rhizosphere (Li et al. 2018). Under AWD, the addition of organic fertilizers notably enhanced N, P, and K absorption by rice, particularly promoting P transfer to panicles and grains, resulting in increased grain weight and yield (Yang et al. 2004). Similar reports showed AWD-grown plants with higher grain yields, improved P transport efficiency, metabolic P efficiency, and P harvest index values (Deng et al. 2021). SOC and N benefits increased with straw, biochar, and slow N addition under AWD, increasing yields by 2–3% (Zhang et al. 2023a). AWD combined with compound N reduces paddy runoff and N leaching (Qi et al. 2023). Additionally, AWD and biochar together raise yields by approximately 15%, improving photosynthesis rate, and N, P, and K efficiency during the nutrition and maturation stages (Haque et al. 2022; Deng et al. 2021).

Microbes transform inorganic mercury to toxic methylmercury (MeHg), which then accumulates in rice (Tanner et al. 2018; Guo et al. 2021). AWD effectively reduces MeHg in rice ecosystems (Table 3). AWD at 35% soil moisture curtails surface water MeHg by 68% and 39% during the growth and fallow seasons, respectively, decreasing rice grain MeHg and total mercury by 60% and 32%, respectively (Tanner et al. 2018). Over a 2 year period, AWD lowered sulfur (S) (4% and 15%), and arsenic (As) (14% and 26%) in grains, while elevating manganese (Mn) (19% and 28%), copper (Cu) (81% and 37%), cadmium (Cd) (28% and 67%) (Norton et al. 2017). This aligns with AWD lowering As, raising Cd, Cu, selenium (Se), and Zn in rice (Martínez-Eixarch et al. 2021). Drying severity in AWD lowers the amounts of total and inorganic As in grains, while lowering inorganic As content requires lower SWP (Norton et al. 2017; Carrijo et al. 2022). Grain Cd hinges on AWD timing, where early reproductive AWD raises Cd, and AWD during multiple stages can elevate it even further (Norton et al. 2017; Carrijo et al. 2022). Combining AWD with P application lowers the amounts of heavy metal elements, like Pb and As, in grains (Song et al. 2021a; Wu et al. 2021). Yet, AWD weakens rice's sodium (Na) tolerance. Alkaline soil AWD yields are lower due to high levels of exchangeable Na causing impermeable layers, making roots draw deeper water (Carrijo et al. 2017; Yang et al. 2004). Also, high Na causes toxicity, and in non-flooded soil, it's absorbed to a greater extent, affecting AWD-associated Na poor tolerance (Carrijo et al. 2017).

Rice yields about 10% of global agricultural GHG emissions, largely from CH_4_ in flooded fields (Kraus et al. 2022). AWD reduces GHG emissions and water use (Carrijo et al. 2017; Martínez-Eixarch et al. 2021; Kraus et al. 2022). Applying AWD in rice cultivation reduces water use by 30% and methane emissions by 30–70% while maintaining yield (evidence from Bangladesh et al. 2022). Mild AWD reduces cumulative CH_4_ emissions by 87.1% on average (Liao et al. 2020). A study found AWD could lower annual methane emissions by 51% while still boosting yield by 9% (Arai 2022). Rice under AWD showed reduced CH4 emissions, and increased yield through improved carbohydrate transport (Arai 2022). Also, while AWD lowered CH_4_ emissions by 23%, it increased N_2_O by 8% (Kraus et al. 2022). Increases in N_2_O have been tied to urea application and irrigation methods. Mild AWD raised cumulative N_2_O emissions by 28.8%, and a further 17.9% in conjunction with urea, cutting global warming potential (GWP) by 62.9% (Wu et al. 2022). Some research has suggested that AWD might even cut GWP by 90% (Martínez-Eixarch et al. 2021). Hence, AWD stands as an effective method for sustainable development with strong ecological benefits.

## Unlocking the secrets of survival: exploring a crop's molecular adaptation to drought stress under deficit strategies

### Unveiling molecular regulatory mechanisms: advantages of water-saving irrigation in light of crop responses

**Fig. 4:**
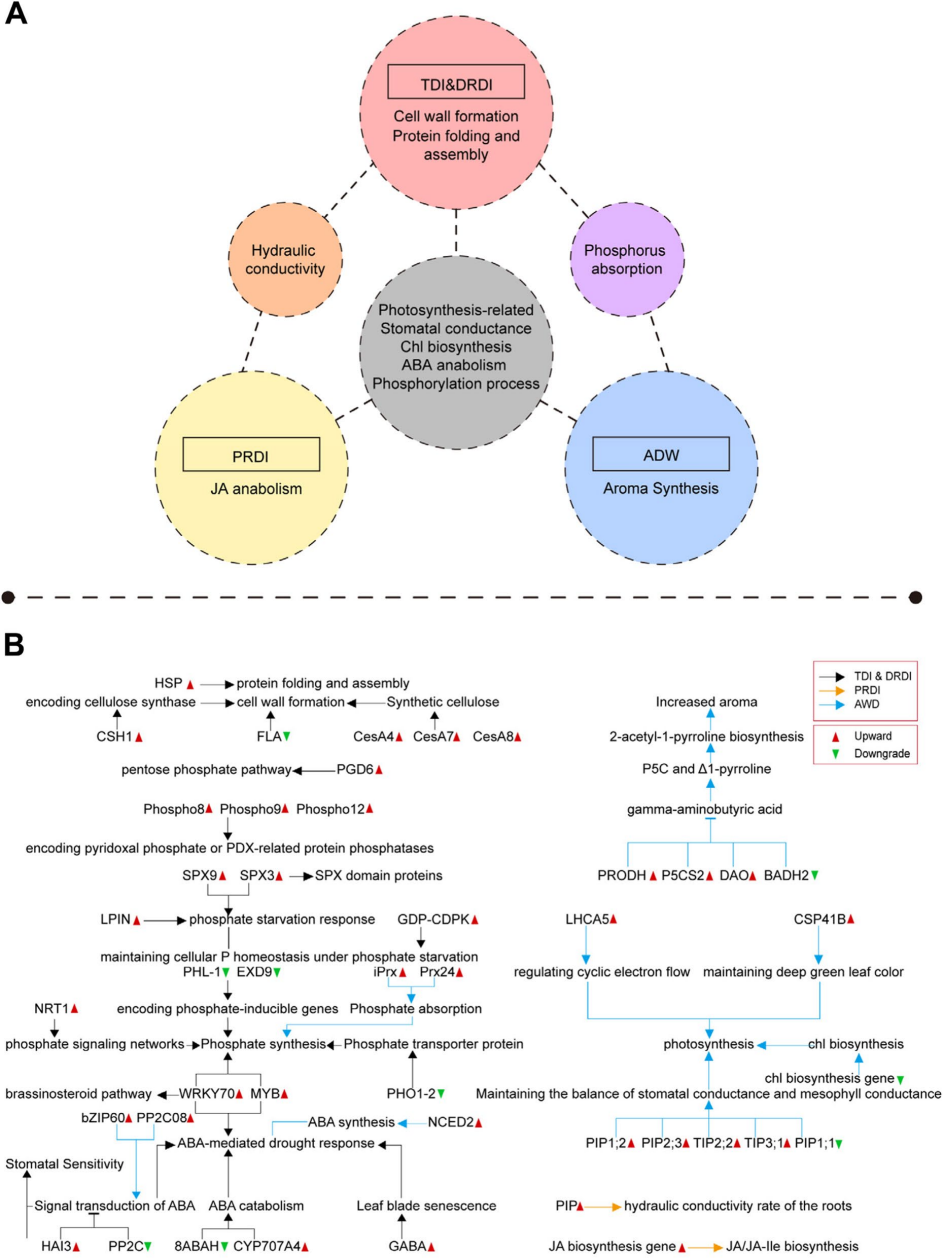
Molecular regulatory mechanisms of crops under different water-saving irrigation techniques. **A** Major metabolic pathways activated by crops to adapt to different water-saving irrigation technologies. **B** Differentially expressed genes or proteins under different water-saving irrigation and their functions. Differentially expressed genes or proteins on different metabolic pathways, black arrows; orange arrows; blue arrows represent the genes and metabolic pathways mentioned in the studies on TDI & DRDI; PRDI: AWD, respectively. Red triangles represent increasing expression, green triangles represent decreasing expression

Application of water-saving irrigation techniques brings about the reduction in water consumption. This is particularly evident in instances of DI strategies, where unavoidable drought stress can impact plants to a certain degree. An essential factor in the successful integration of water-saving irrigation methods for achieving optimal crop production lies in the judicious exploitation of plant drought stress mechanisms. Traditional responses to drought stress encompass an array of physiological reactions, including stomatal closure, root development, cellular adaptation, photosynthesis, the production of ABA and JA, ROS elimination, and the transmission of diverse signaling pathways (Ullah et al. 2017). ﻿Omics technologies have been widely used to study molecular physiological processes and characteristics (Song et al. 2022; Song et al. 2023). Under water-saving irrigation, a multitude of genes exhibit differential expression at the molecular level, orchestrating various metabolic pathways (Fig. 4A). Consistent with traditional drought stress, these pathways encompass signal transduction, oxidation–reduction processes, the synthesis of secondary metabolites, non-biological stress responses, enzyme activities, and the involvement of heat shock proteins, which collectively equip plants to withstand a range of challenges (Lopez et al. 2020; Gregorio Jorge et al. 2020; Chaichi et al. 2019). Analyzing pivotal differentially expressed genes (DEGs) within these metabolic pathways not only enhances our understanding of crop regulation mechanisms under distinct water-saving irrigation treatments (Fig. 4B), but also unveils the potential for uncovering distinctive response mechanisms unique to crops under water-saving irrigation conditions, which are in contrast to conventional drought scenarios.

Under deficient irrigation strategies, both TDI and DRDI hinge on directly triggering the corresponding regulatory mechanisms in plants as a response to diminished water availability. When it comes to technologies aimed at directly reducing water usage, the approach often entails simulating a conventional drought scenario while harnessing the plant's inherent resilience to drought stress. This methodology facilitates the accomplishment of water conservation goals. However, in the cases of PRDI and actual AWD, the activation of regulatory networks isn't solely predicated on water reduction. Instead, it's based on a blend of distinct molecular regulatory mechanisms present in plant roots within dry and wet soils. Nevertheless, the spatial and temporal requisites for the dry-to-wet transitions in PRDI and AWD differ, thereby giving rise to some specific molecular regulatory mechanisms.

Traditionally, under conventional drought conditions, crops predominantly regulate their drought resistance through a combination of ABA-dependent and ABA-independent pathways (Du et al. 2018). For example, many crops, like rice, have evolved drought escape mechanisms to curtail their life cycle under drought circumstances (Du et al. 2018; Chen et al. 2020). In the context of water-saving irrigation, experimental studies have reported a substantial differential gene expression that pertains to molecular functions, biological processes, and cellular components when subjected to reduced water irrigation (Lopez et al. 2020; Gregorio Jorge et al. 2020). This concurs with the conventional drought response, wherein genes linked to ABA synthesis and degradation take the spotlight as significant features for crops facing drought stress. Furthermore, under water-saving irrigation, studies have found that concerning TDI and DRDI, genes involved in ABA responses, such as *PP2C* (*Phvul.001G021200*) and a gene with a putative ABA 8'-hydroxylase (*Phvul.002G122200*), showcase inhibition under drought conditions (Lopez et al. 2020). *PP2C* stands as a pivotal negative regulatory factor in ABA signal transduction, whereas *Phvul.002G122200* is associated with ABA catabolism (Cutler et al. 2010). Conversely, under AWD conditions, genes like ABA biosynthesis gene *OsNCED2* and signal transduction genes (*OsbZIP60*, *OsPP2C08*) were upregulated (Song et al. 2020). *OsPP2C08 (Os01g0656200),* belonging to the A subfamily of the rice OsPP2C family, actively participates in the ABA signaling pathway while responding to environmental cues (Xue et al. 2008). Remarkably, rice *OsPP2C08* displayed an approximate 22-fold increase under AWD due to drought stress, and its counterpart in Arabidopsis (AtPP2CA enzyme) also plays a role in drought stress (Song et al. 2020; Yoshida et al. 2006). Furthermore, research has illuminated the upregulation of the HIGHLY ABA-INDUCED PP2C GENE 3 *(HAI3*) and the soybean-specific hub gene *CYP707A4* in response to ABA (Fang, et al. 2023). *HAI3*, which functions as an ABA signal suppressor gene, contributes to the early activation and heightened sensitivity of stomatal control (Fang, et al. 2023). On a different note, *CYP707A4* represents an ABA degradation gene associated with transpiration rate (Fang, et al. 2023). Its upregulation under water-deficient conditions is regarded as a fine-tuning mechanism in plants, allowing for the maintenance of higher stomatal conductance (gm) through ABA degradation (Fang, et al. 2023). The mechanism where plants activate downstream regulation through ABA has been validated through earlier studies on plant responses to drought stress. For instance, in rice, the OsASR5 protein has been demonstrated to regulate ABA biosynthesis and encourage stomatal closure as a response to combat drought stress (Li et al. 2017b). Moreover, the mechanism wherein ABA mediates the abbreviation of a plant's life cycle also stands as a pivotal element for the feasibility of implementing water-saving irrigation for crops. Taking rice as an example, the onset of early drought stress in rice development sparks the accumulation of ABA, which, in turn, exerts regulatory control over numerous flowering-related genes to advance early flowering (Du et al. 2018). Throughout this process, photoreceptors, components of the circadian rhythm, and genes linked to flowering, such as *OsTOC1*, *Ghd7*, and *PhyB*, are recognized to participate in ABA-dependent drought stress responses (Du et al. 2018). The strategy of expediting flowering to hasten the life cycle offers an avenue to earlier crop harvesting, underscoring one of the capacities of water-saving irrigation techniques to enhance agricultural productivity. Beyond ABA, the WRKY transcription factor has been documented as a negative regulator of plant aging (Besseau et al. 2012), and is implicated in steering plant growth and responding to drought through the brassinosteroid pathway (Chen et al. 2017a). Research has revealed that, under water-saving irrigation, the expression of *WRKY70* (*Phvul.008G185700*) transcription factors experiences an upsurge during drought stress (Lopez et al. 2020). Furthermore, studies have highlighted that two soybean GABA transporter 1-encoding genes, implicated in regulating leaf senescence, undergo a significant upregulation during moderate or severe drought conditions in plants (Fang, et al. 2023). Hence, the simulation of drought conditions by water-saving irrigation to activate crop ABA synthesis and senescence mechanisms stands as a critical determinant for maintaining crop yield. In this aspect, water-saving irrigation draws parallels with traditional drought stress.

Simultaneously, keywords like drought, ABA, and senescence are intricately link with various biological synthesis and signal transduction processes. Among these, MYB transcription factors regulate the biosynthesis of secondary metabolites and mediate a plant's adaptability to abiotic stressors, including drought (Chen et al. 2021b). In previous studies, *GmMYB14* has been reported to regulate plant architecture, high-density yield, and drought tolerance in soybeans through the brassinosteroid (BR) pathway (Chen et al. 2021b). This results in reduced plant height, internode length, leaf area, petiole length, and petiole angle, while increasing high-density yield under field conditions (Chen et al. 2021b). Similarly, in rice, there have been reports of the OsFTIP6-OsHB22-OsMYBR57 module regulating drought response (Yang et al. 2022). In this module, OsMYBR57, a MYB-related protein, directly regulates the expression of several key drought-related *OsbZIP* genes to respond to drought treatment (Yang et al. 2022). There are also reports suggesting that MYB-related transcription factors can enhance plant tolerance to biotic stress through the C-repeat/dehydration-responsive element binding proteins (CBF/DREB) and ABA signaling pathways (Chen et al. 2023). CBF/DREB plays a crucial role in abiotic stress responses, and in wheat, it has been found that drought can induce the activity of the promoters of two DREB/CBF genes, *TaDREB3* and *TaCBF5L*, thereby improving plant stress tolerance and maintaining yield (Yang et al. 2020a). Furthermore, MYB (*Phvul.003G028000)* is not only involved in crop drought tolerance but also indirectly participates in phosphate synthesis (Lopez et al. 2020). In wheat, deficient irrigation has been reported to significantly upregulate DEGs encoding 6-phosphogluconate dehydrogenase (PGD6) (Ma et al. 2019). Increased accumulation of PGD6 enhances the efficiency of the pentose phosphate pathway, providing more nicotinamide adenine dinucleotide phosphate (NADPH), precursors, or cofactors for biosynthesis. This enhances plants’ ability to withstand drought conditions (Ma et al. 2019; Chen et al. 2004). In addition to ABA signaling, the Mitogen-Activated Protein Kinase (MAPK) cascade is a key strategy that plants have developed to respond to various biotic and abiotic stresses. It participates in signal transduction from extracellular stimuli and regulates cellular responses (Group and M. 2002). The MAPK cascade consists of at least three different protein kinases: MAPKKK, MAPKK, and MAPK, which activate in sequence through phosphorylation (Group and M. 2002). Drought stress induces the expression of stress-related transcription factors and genes, such as those involved in ROS clearance, ABA, or MAPK signaling. These genes activate various drought-related pathways, thereby inducing plant tolerance to drought stress (Ullah et al. 2017). In cotton, the nuclear-localized and membrane-localized MAPK cascade pathway GhMAP3K62-GhMKK16-GhMPK32 targets and phosphorylates the nuclear-localized transcription factor GhEDT1 to activate downstream *GhNCED3* (Chen et al. 2022). This mediates ABA-induced stomatal closure and drought response (Chen et al. 2022). These signals, targeted at the nuclear-localized transcription factor *GhEDT1*, cause a cascade that culminates in the downstream activation of *GhNCED3* (Chen et al. 2022). Additionally, in practical agricultural settings, the upregulation of genes in the MAPK signaling pathway has been found to enhance drought resistance in crops. For instance, *GhMKK3* enhances drought tolerance in cotton (Wang et al. 2016), while *GhMKK1* is involved in the enhancement of salt and drought tolerance (Lu et al. 2013). Therefore, drought stress under water-saving irrigation can induce a series of signal transduction and biosynthesis processes in crops, that increase their drought tolerance, adjust plant structures, and potentially maintaining or even increasing yield.

Moreover, the cell wall remodeling is an often-observed response in plants faced with the challenge of drought stress (Gregorio Jorge et al. 2020; Ezquer et al. 2020; Gall et al. 2015). Under deficient irrigation, certain DEGs participate in cell wall modification. For instance, two genes encoding cellulose synthase H1 (*Phvul.005G117833* and *Phvul.005G116501*) are upregulated in samples subjected to drought treatment, while a gene encoding an extension protein (*Phvul.004G161500*) is downregulated (Lopez et al. 2020). Secondary cell walls (SCWs) contribute to improving plant drought tolerance by alleviating osmotic disturbances caused by drought (Gregorio Jorge et al. 2020). The cellulose synthase complex (CSC), primarily responsible for cellulose synthesis in SCWs, is predominantly composed of CesA4, CesA7, and CesA8 proteins (Gregorio Jorge et al. 2020; Hall et al. 2017). Interestingly, all core components of the CSC are found among the upregulated genes (Gregorio Jorge et al. 2020). Coincidentally, research has also revealed a significant downregulation of multiple *FASCICLIN-LIKE ARABINOGALACTAN (FLA)* genes in soybeans. These *FLA* genes were previously reported to be associated with cell proliferation and cell wall structure formation (Fang, et al. 2023; MacMillan et al. 2010). In rice, an interesting finding was made regarding the rice phytochrome-interacting factor-like protein OsPIL1/OsPIL13, which was identified as a crucial regulator of reduced internode elongation under drought conditions (Todaka et al. 2012). *OsPIL1* promotes internode growth, and genes downstream of *OsPIL1* are enriched in cell wall-related genes responsible for cell growth (Todaka et al. 2012). *OsPIL1* plays a key regulatory role in reducing plant height in response to drought stress through cell wall-related genes (Todaka et al. 2012). Under drought conditions, the expression of *OsPIL1* is inhibited during the photoperiod (Todaka et al. 2012). This highlights the necessity of applying water-saving irrigation based on actual conditions and selecting suitable seasons to meet the crop's requirements for light and temperature. This also explains why the effects of using water-saving irrigation techniques can vary depending on the timing of application. Furthermore, heat shock proteins (HSPs) in plants facilitate protein folding or assembly under stress conditions (Driedonks et al. 2015; Swindell et al. 2007), enhancing plant tolerance to drought and high temperatures (Burke and Chen 2015; Cho and Hong 2006). Research has indicated that a member of the HSP70 family, *GhHSP70-26*, is involved in the response of cotton to drought stress (Guo et al. 2023). The relative expression level of *GhHSP70-26* shows a linear correlation with the comprehensive drought resistance of cotton seedlings (Guo et al. 2023). In rice, the HSP90 family gene *OsHSP50.2* has been found to have increased transcription levels in response to both heat and osmotic stress (Xiang, et al. 2018). This leads to reduced electrolyte leakage and malondialdehyde levels in rice, along with a smaller reduction in chlorophyll and higher SOD activity, demonstrating greater drought resistance (Xiang, et al. 2018). However, the expression of HSPs is rapid and transient, so after an extended period of drought, HSPs and genes involved in protein folding are downregulated, indicating that these proteins are no longer needed (Gregorio Jorge et al. 2020).

In addition to utilizing traditional drought response mechanisms to enhance drought tolerance and maintain yield, specific regulatory mechanisms have also been identified in PRDI and AWD. For instance, under most drought stress conditions, PIP-related genes significantly increase plant growth rate, transpiration rate, stomatal density, and photosynthetic efficiency (Chen et al. 2021c; Aharon et al. 2003). However, enhanced symplastic water transport through the aquaporins facilitated by membrane water channel proteins under drought stress might have detrimental effects (Chen et al. 2021c; Aharon et al. 2003). As a result, PIP-related genes in plants are often downregulated. Yet, studies have found that PRDI can induce specific responses to enhance the water uptake rate of the hydrated root, which is believed to be regulated by signals produced by the leaves (Luo et al. 2019; McLean et al. 2011; Pérez-Pérez et al. 2020). In cotton, the hydraulic conductivity rate (L) of roots and the water absorption in hydrated roots may be the result of increased expression of the intrinsic protein gene (*PIP*) (Luo et al. 2019; McLean et al. 2011). Moreover, the contents of jasmonic acid (JA) and jasmonic acid-isoleucine conjugate (JA-Ile), and the expression of three JA biosynthesis genes in the leaves of PRDI plants are increased (Luo et al. 2019). Although the expression of the three JA genes in the roots does not change, the JA/JA-Ile content increases (Luo et al. 2019). Therefore, under PRDI conditions, plants can induce the expression of genes related to leaf JA synthesis, synthesize more JA/JA-Ile, and transfer them to the roots through the cortex to induce the expression of *GhPIP*, thereby increasing the hydraulic conductivity rate of the roots (Luo et al. 2019; McLean et al. 2011; Han et al. 2023). Under AWD conditions, aside from the significant differential expression of genes related to ABA synthesis and metabolism, substantial differential expression of genes associated with photosynthesis contributes to rice's yield enhancement under AWD (Song et al. 2020). Transcriptomic analysis has revealed that these DEGs are mainly related to Chl, light-harvesting complexes (LHCs), PSI, and PSII (Song et al. 2020). Similar to traditional drought stress, AWD also leads to the downregulation of gene expression in pathways related to chlorophyll synthesis and other aspects of photosynthesis (Song et al. 2020). When rice was subjected to AWD irrigation treatment, 14 DEGs involved in Chl biosynthesis were downregulated in the flag leaves, and the enzymes encoded by these DEGs were important in the Chl biosynthesis pathway (Song et al. 2020). Chl plays a vital role in capturing sunlight and converting it into chemical energy, and any interference with the Chl concentration will lead to changes in photosynthesis (Vandoorne, et al. 2012). Secondly, the genes involved in LHCs were downregulated in AWD (Song et al. 2020). The primary function of the light-harvesting complex is to capture solar energy and transfer the excited energy to the reaction center (Masuda et al. 2002). Thirdly, the DEGs involved in photosynthetic pathways such as PSI, PSII, cytochrome b6/f complex, photosynthetic electron transfer, and F-type ATP synthase were downregulated in the flag leaves, affecting the synthesis metabolism of key components in the photosynthetic pathway (Song et al. 2020). However, more interesting was that under the severe drought conditions caused by the AWD irrigation technique, several genes in rice, namely, *OsPIP1;1*, *OsPIP1;2*, *OsPIP2;3*, *OsTIP2;2*, and *OsTIP3;1*, play a crucial role in positively regulating Gs and gm in rice plants (He et al. 2021). Maintaining a high gm is one of the primary factors for sustaining high photosynthesis in rice under water stress (He et al. 2022). The *OsPIP1;1* gene shows a clear positive correlation with Gs and gm, and its relative expression level significantly decreases under AWD conditions. Conversely, *OsPIP1;2*, *OsPIP2;3*, *OsTIP2;2*, and *OsTIP3;1* are all upregulated to mitigate the sharp decline in Gs and gm during severe drought (He et al. 2021; Bai et al. 2021). Furthermore, it has been reported that AWD enables rice to maintain a high expression of *OsLHCA5* and *OsCSP41B* genes under water stress (He et al. 2022). LHCA5 is an essential component of the chloroplast NADH dehydrogenase super complex and plays a critical role in regulating cyclic electron flow (Kato et al. 2018). Additionally, the *OsLHCA5* gene indirectly influences ATP and NADPH content in the PSI system (Selmar and Kleinwächter 2013). The *CSP41B* gene, located in the chloroplast, contributes to maintaining a deep green leaf color in crops (Mei et al. 2017). The *OsLHCA5* and *OsCSP41B* genes are candidate genes that co-regulate gm and energy distribution to achieve high photosynthesis under severe water stress (He et al. 2022). The *OsLHCA5* and *OsCSP41B* genes are candidate genes that co-regulate gm and energy distribution to achieve high photosynthesis under severe water stress (Li et al. 2017b), the drought stress induced in rice by AWD allows for the maintenance of high gm and efficient energy distribution (He et al. 2022). This enables rice to sustain high photosynthetic activity, and it's one of the reasons why rice can maintain high yields under AWD conditions. Moreover, studies have revealed that under AWD treatment, rice can enhance enzyme activity by inducing the expression of genes such as *PRODH*, *P5CS2*, and *DAO*, while inhibiting other pathways, such as BADH2, to downregulate gamma-aminobutyric acid (Bao et al. 2018). This leads to the upregulation of P5C and Δ1-pyrroline synthesis and promotes 2-AP biosynthesis, leading to improved rice quality (Bao et al. 2018). Regarding the promotion of P uptake in rice under AWD treatment, it has been found that AWD significantly enhances the activity of ionic-bonded cell-wall-located class III peroxidases (iPrx) and the expression of *OsPrx24* (a gene encoding iPrx) (Acharya et al. 2016; Yang et al. 2020b). iPrx is involved in the clearance of ROS in the cell wall (Acharya et al. 2016; Yang et al. 2020b). *OsPrx24* is primarily expressed in the root epidermis and participates in the formation of infection structures by regulating iPrx activity and H_2_O_2_ concentration under AWD, thereby facilitating phosphorus absorption (Acharya et al. 2016; Yang et al. 2020b). These are unique regulatory mechanisms under AWD. Therefore, understanding the similarities and differences in the regulatory mechanisms of crops under water-saving irrigation and traditional drought can further help harness the potential of crops and water-saving irrigation techniques, with the aim to create greater agricultural value.

### Unlocking the potential of plants: using genetic modification and water-saving technologies

Understanding the molecular adaptability of crops to drought stress under DI strategies contributes to the breeding, selection, and introduction of actual or potential water-saving varieties to fully exploit the drought resistance potential and superior performance of crops. Currently, many excellent new varieties have been developed that are better adapted to water-saving irrigation. The combination of both, not only harnesses the inherent performance of crops, but also maximizes the advantages of water-saving irrigation. Research has shown that silencing the *OsSYT-5* gene enhances the drought tolerance of rice (Shanmugam et al. 2021). Transgenic plants where this gene has been supressed exhibit higher photosynthetic rates, lower gm, and transpiration under water-deficit conditions, and increased ABA content in both drought and normal conditions, resulting in delayed drought stress symptoms, higher pollen vitality, and increased grain production compared to the wild type (Shanmugam et al. 2021). Overexpression lines of the galactinol synthase 2 gene (*OsGolS2*) were found to enhance leaf water content, maintain higher photosynthetic activity, reduce the rate of plant growth deceleration, and exhibit stronger recovery capabilities (Selvaraj et al. 2017). Additionally, overexpression of *OsHAK1* in rice seedlings enhances their drought tolerance compared to the wild type, while the *OsHAK1* knockout mutants display lower tolerance to stress during both nutritional and reproductive stages, and exhibited delayed growth (Chen et al. 2017b). Compared to wild-type seedlings, the lipid peroxidation level was lower, the activity of antioxidant enzymes was higher, and the accumulation of proline was higher in *OsHAK1* overexpressing rice, demonstrating higher drought resistance and 35% higher grain yield under drought conditions (Chen et al. 2017b). This greatly enhances the adaptability of rice to water-saving irrigation. Moreover, research has suggested that *OsJAZ9* plays a crucial role in conferring drought tolerance in rice by affecting JA and ABA signal transduction (Singh et al. 2021). Overexpression of *OsJAZ9* in rice increases ABA and JA content, improves osmotic pressure, reduces leaf width and stomatal density, decreases leaf transpiration rate, and exhibits better drought resistance compared to the wild type (Singh et al. 2021). In wheat, which is particularly susceptible to water deficiency during the jointing stage of development, sucrose non-fermenting 1-related protein kinase 2 (SnRK2) acts as a key signaling hub in response to drought stress (Zhang, et al. 2023). Overexpression of SnRK2 in wheat leads to drought-resistant varieties, and studies have shown that ectopic expression of SnRK2 in rice also imparts higher drought tolerance (Zhang, et al. 2023). Research has also explored the performance of different wheat genotypes under various levels of water-saving irrigation (Liu et al. 2016b). The results of which indicate that Shijiazhuang 8, a drought-resistant variety, maintains a higher net photosynthetic rate, gm, and transpiration under drought stress, resulting in higher grain yield and WUE compared to Yanmai 20, a drought-sensitive variety (Liu et al. 2016b). The isopentenyltransferase gene (*IPT*), which encodes a rate-limiting enzyme in cytokinin biosynthesis, significantly improves the drought resistance of cotton, especially during the pre-flowering (nutritional) stage under water deficit stress (Zhu et al. 2018). Overexpression of *IPT* in cotton plants demonstrates higher drought tolerance and yield compared to control plants (Zhu et al. 2018). *LOS5/ABA3 (LOS5),* encoding a molybdenum co-factor, is essential for activating aldehyde oxidase involved in ABA biosynthesis (Yue et al. 2012). Overexpression of AtLOS5 in cotton plants enhances ABA production, ABA-induced physiological regulation, improves antioxidant enzyme activity, and significantly improves membrane integrity under water deficit stress (Yue et al. 2012). In maize, genotypes with partial stomatal closure in response to elevated atmospheric vapor pressure deficit exhibit higher drought resistance and higher yield under water deficit conditions (Jafarikouhini et al. 2022). Studies have also suggested that maize plants with overexpression of the zeaxanthin epoxidase gene (*ZEP*) are more sensitive to water deficit (Borel, et al. 2001). In soybean, it has been found that soybean lines with higher expression of *GmWRKY106* and *GmWRKY149* genes show stronger drought tolerance under water deficit conditions (Dias et al. 2016). Overexpression of *GmDREB2A* and *GmDREB2A* in transgenic plants leads to higher leaf β-glucose and fructose concentrations, indicating greater drought resistance (Marinho et al. 2019). In conclusion, whether through hybridization or conferring individual gene functionality, the support of molecular regulatory mechanism theories is required. Understanding the molecular adaptability of crops contributes to the selection of drought-resistant crop varieties and the better integration of water-saving irrigation, thereby creating more suitable agricultural irrigation and production systems and generating higher economic and ecological value.

## Conclusions and perspectives

This review underscores the significance of selecting appropriate irrigation technologies based on the desired water consumption reduction, improved WUE, and sustained crop yield in sustainable agriculture. DRFI is suitable for most crops other than paddy fields, improving WP, stimulating plant root growth, and increasing fertile spikelets, with a high potential for yield increase. TDI has a high water-saving potential, which improves WP, but can affect plant yield through various mechanisms. DRDI significantly enhances WP based on DRFI, but its yield-increasing potential is moderate and requires proper nutrient management. PRDI improves water use efficiency, particularly promoting root development and biomass production under dry conditions, and also influences soil microorganisms. AWD is suitable for paddy crops, enhancing WP with minimal yield impact, and can regulate harmful ion concentrations and greenhouse gas emissions. Each technique offers specific advantages and disadvantages, and agricultural practitioners can use this review as a guide to determine the most suitable irrigation method for their circumstances.

Furthermore, there are a variety of methods that can be used in future research to further realize the potential of water-saving irrigation techniques, including the integration of multiple irrigation techniques to maximize water conservation and crop performance, such as incorporating drip irrigation to supplement PRDI or utilizing a root zone irrigation strategy for AWD. Additionally, exploring the use of innovative materials for irrigation infrastructure, such as integrating biodegradable mulches or smart irrigation membranes into water-saving irrigation technologies, could contribute to more sustainable and eco-friendly practices. Furthermore, research efforts should focus on understanding the molecular regulatory mechanisms underlying crop responses to different water-saving techniques. Many studies have already focused on exploring the functions of genes to cultivate drought-resistant and water-efficient crop varieties. Integrating these varieties into water-saving irrigation techniques can greatly enhance the water-saving potential of both approaches. By pursuing these avenues of research and innovation, we can address the challenges of water scarcity and food security while promoting a sustainable and resilient agricultural sector for the future.

## Supplementary Information

**Additional file 1. Figure S1. **Web of science core collection database keyword analysis from January 2012 to April 2023 and patterns of water-saving irrigation techniques. A: Keyword co-occurrence network analyzed by BibExcel and VOSviewer. Node colors represent modularity, node size represents how often keywords appear. B: Burst keyword analysis. The length of colored boxes represents burst status duration. Colors represent burst strength. The keywords "treatment" and"mission" are diluted by a factor of 20 and 5, respectively, due to the large difference in values with the other keywords in the figure. C: Statistical table of relevant publications from 2012 to 2022. The horizontal coordinate represents the year and the vertical coordinate represents the frequency of keywords. 

## Data Availability

No datasets were generated or analysed during the current study.
