# Supplementary material for: Water-saving techniques: physiological responses and regulatory mechanisms of crops
Source: Adv Biotechnol (Singap). 2023 Oct 26;1(4):3. doi: 10.1007/s44307-023-00003-7 (PMC11727597; doi:10.1007/s44307-023-00003-7)
Supplement: Supplementary file 1 — Additional file 1. Figure S1. Web of science core collection database keyword analysis from January 2012 to April 2023 and patterns of water-saving irrigation techniques. A: Keyword co-occurrence network analyzed by BibExcel and VOSviewer. Node colors represent modularity, node size represents how often keywords appear. B: Burst keyword analysis. The length of colored boxes represents burst status duration. Colors represent burst strength. The keywords "treatment" and"mission" are diluted by a factor of 20 and 5, respectively, due to the large difference in values with the other keywords in the figure. C: Statistical table of relevant publications from 2012 to 2022. The horizontal coordinate represents the year and the vertical coordinate represents the frequency of keywords. [file 44307_2023_3_MOESM1_ESM.docx]

**Figures**

**
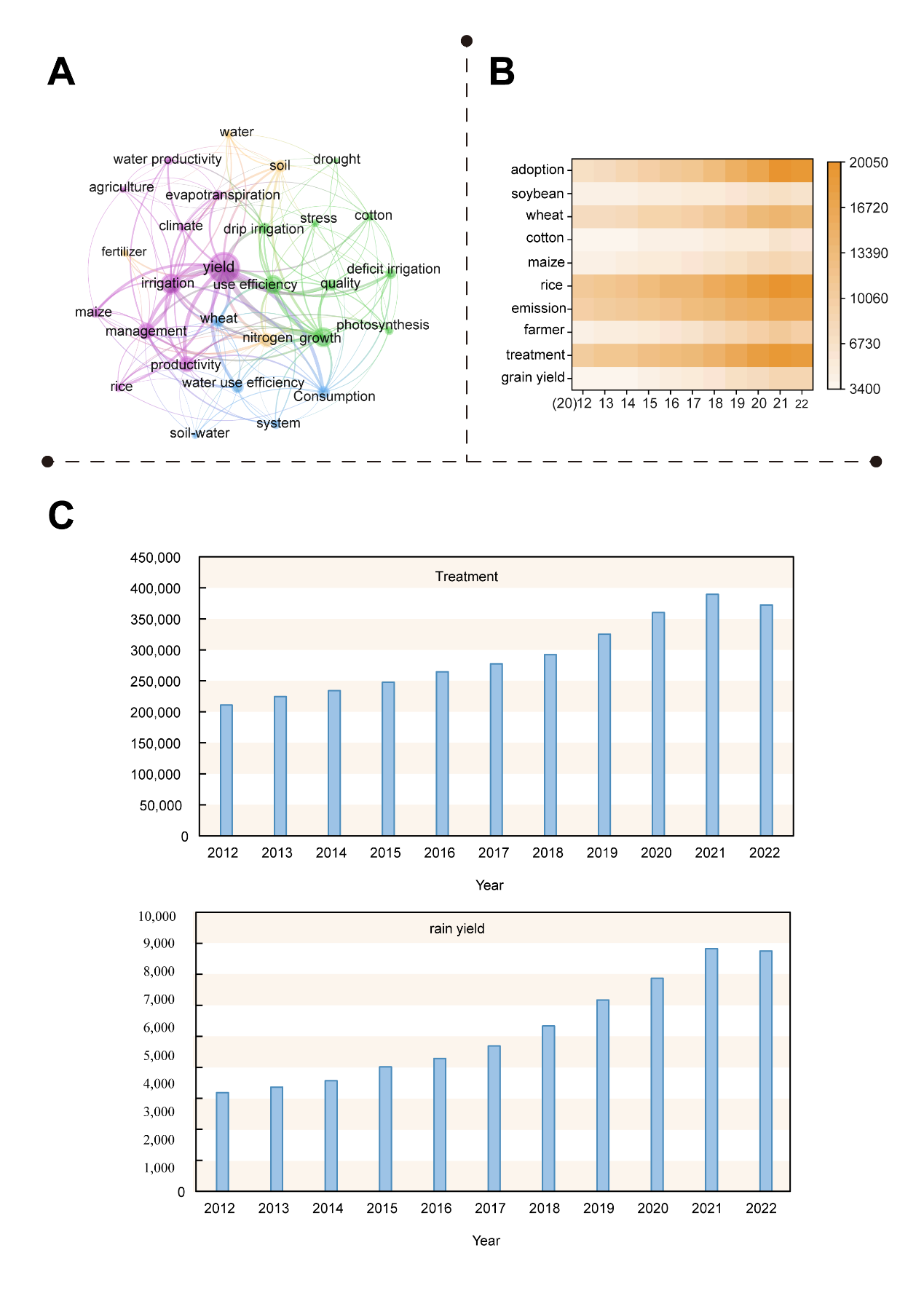
**

Figure S1: Web of science core collection database keyword analysis from January 2012 to April 2023 and patterns of water-saving irrigation techniques. A: Keyword co-occurrence network analyzed by BibExcel and VOSviewer. Node colors represent modularity, node size represents how often keywords appear. B: Burst keyword analysis. The length of colored boxes represents burst status duration. Colors represent burst strength. The keywords "treatment" and "mission" are diluted by a factor of 20 and 5, respectively, due to the large difference in values with the other keywords in the figure. C: Statistical table of relevant publications from 2012 to 2022. The horizontal coordinate represents the year and the vertical coordinate represents the frequency of keywords.
